# European public health best practice portal - process and criteria for best practice assessment

**DOI:** 10.1186/s13690-022-00892-5

**Published:** 2022-05-06

**Authors:** Magdalena Stepien, Ingrid Keller, Marianne Takki, Sandra Caldeira

**Affiliations:** 1 Joint Research Centre, European Commission, Via E. Fermi, 2749, I-21027 Ispra (VA), Italy; 2 Health Security Unit, Directorate-General for Health and Food, European Commission, 11, rue Eugène Ruppert, L-2453 Luxembourg, Luxembourg; 3 Health promotion, disease prevention, financial instruments Unit, Directorate-General for Health and Food Safety, European Commission, 11, rue Eugène Ruppert, L-2453 Luxembourg, Luxembourg

## Abstract

**Background:**

Non-communicable diseases (NCDs) are major and growing burden on population health and the use and cost of healthcare in EU Member States and beyond. Different countries face many common challenges in public health and can learn from each other. The exchange of ‘best practices’ is one way to tackle the observed disparities in health sector. To address the United Nations Sustainable Development Goals, the European Commission developed the EU Public Health Best Practice Portal to facilitate the exchange of best practices and facilitate their implementation in other EU countries or regions. The ultimate aim of the portal is to reduce NCDs burden and the prevalence of their risk factors by promoting implementation and scale up of evidence-based effective interventions in the areas of health promotion, disease prevention and management of NCDs.

**Results:**

This article presents the rationale and the process, ranging from best practice assessment to their transfer to interested Member States, applied in the EU Public Health Best Practice Portal. The portal selects best practices using rigorously defined criteria for best practice assessment. This article further provides an overview of other similar initiatives in Europe and internationally that collect and disseminate information on interventions and actions to combat NCDs.

**Conclusion:**

Exchange of best practices is a promising tool in tackling NCDs. Transfer and scaling up of policies and interventions between countries may contribute to tackle disparities observed between countries in regards to the prevalence of risk factors and associated diseases.

## Background

Diseases of the circulatory system and cancers, belonging to the group of non-communicable diseases (NCDs), were responsible for over half of all deaths in Europe in 2017 [1]. About two-thirds of premature deaths under age 75 could have been prevented, most of which were considered preventable through effective public health measures, including primary prevention interventions [1]. Additionally, considering the fact that COVID-19 pandemic outbreak considerably affected especially those with underlying chronic health conditions [2], the work in the area of NCDs control is of increased importance.

The public health interventions for NCDs prevention include a variety of tools that in most cases aim to reduce mortality through risk factor modification [3]. A public health measure that produces desirable outcomes in improving health in real-life settings and which can be adopted elsewhere can be acknowledged as a “best”, “good” or “promising” practice. A best practice should show evidence of effectiveness and efficiency, possible replicability in another setting, sustainability, ethical soundness, relevance, and community and stakeholder participation [4].

The European Commission’s approach on NCDs focuses on prevention across sectors and policy fields, combined with efforts to strengthen health systems. This is not about creating new silos between ‘diseases’ or ‘conditions’, but to enable a comprehensive framework, which can address a wide range of issues from health promotion and disease prevention at population level to more focused interventions where needed. The Article 168 of the EU Treaty on the Functioning of the European Union, referring to public health states that: “The Commission may, in close contact with the Member States, take any useful initiative to promote such coordination, in particular initiatives aiming at the establishment of guidelines and indicators, the organisation of exchange of best practice, and the preparation of the necessary elements for periodic monitoring and evaluation” [5]. With this in mind and to addresses the United Nations Sustainable Development Goals, in particular target 3.4 which states “By 2030, reduce by one third premature mortality from non-communicable diseases through prevention and treatment”, in summer 2018 the European Commission established the European Public Health Best Practice Portal. This online portal collects, assesses and disseminates best practices in health promotion, disease prevention and management of NCDs (the website can be accessed with this link: https://webgate.ec.europa.eu/dyna/bp-portal/) with the ultimate goal to make progress in health promotion and in disease prevention in Europe.

The European Commission through the Public Health Best Practice portal has been collecting best practices in the area of NCDs prevention and health promotion since 2018. The portal is managed and operated by the Directorate General for Health and Food Safety (DG SANTE) and accepts submissions of relevant interventions, implemented at national or regional level, with the view of transfer of what worked elsewhere to another European country.

The portal consists of a submission portal, a database of best practices and detailed information on “marketplace” workshops on selected practices, as well as up-to-date information on the best practices selected by Member States for transfer. In addition, the user can find relevant links to the other national EU best practice portals and other tools such as the Health Promotion and Disease Prevention Knowledge Gateway [6]. Although the main focus of the portal is health promotion and NCDs primary prevention, the portal’s scope extends to secondary prevention and management of disease. It includes practices related to a range of topics from disease determinants (e.g. nutrition, alcohol consumption, tobacco smoking, physical inactivity, and environmental risk factors, to medical conditions (obesity, hypertension, diabetes mental health), etc., and now is also broadening its content to other diseases, including cardiovascular disease, chronic respiratory disease and neurological disorders.

The exchange of best practices has potential to improve health by demonstrating what interventions worked well in similar settings and populations, it avoids “re-inventing the wheel” in designing and piloting of similar interventions, building upon ones’ expertise and more efficient use of resources. We therefore describe the EU portal here to inspire Public Health institutions, but also to increase the visibility of similar portals in Europe. The evaluation criteria and the process we have applied to assess and subsequently transfer practices that were judged as “best” are presented in detail in this article.

### Other best practice portals in Europe

Aside from the EU Best Practice portal, there are a number of other best practice portals within the health domain in the EU (Table 1). The European Monitoring Centre for Drugs and Drug Addiction (EMCDDA) hosts a portal in the areas of prevention, treatment, harm reduction and social reintegration in drug and alcohol users. EMCDDA's website integrates the information ranging from briefings and evidence database (synthesis of systematic reviews, recommendations) to practice registries of evidence-based programmes (including manuals and implementation experiences). The evidence-based recommendations highlight what works, but also what does not (‘Evidence of ineffectiveness).

**Table 1:** Examples of the national best practice portals in EU countries

Portal’s name	Website of the best/good/promising practice portal	Country
The European Monitoring Centre for Drugs and Drug Addiction (EMCDDA) best practice portal	http://www.emcdda.europa.eu/best-practice_en	European
The European Agency for Safety and Health at Work (EU-OSHA) Healthy Workplaces Good Practice Awards	https://osha.europa.eu/en/publications/good-practice-awards-flyer/view	European
Praxisdatenbank Gesundheitliche Chancengleichheit (database of health promotion projects)	https://www.gesundheitliche-chancengleichheit.de/praxisdatenbank/	Germany
Leefstijlinterventies (Lifestyle interventions)	https://www.loketgezondleven.nl/leefstijlinterventies	The Netherlands
PRO.SA Banca dati di progetti e interventi di prevenzione e promozione della Salute (Database of projects and interventions in health promotion and disease prevention)	https://www.retepromozionesalute.it/	Italy
Portal for the exchange of examples of good practice in the field of public health	https://www.nijz.si/publikacije/merila-za-vrednotenje-intervencij-na-podrocju-javnega-zdravja	Slovenia
Profibaza (Database of health interventions)	https://profibaza.pzh.gov.pl/	Poland
Répertoire des interventions efficaces ou prometteuses en prévention et promotion de la santé (Directory of effective or promising interventions in prevention and health promotion)	https://www.santepubliquefrance.fr/a-propos/services/interventions-probantes-ou-prometteuses-en-prevention-et-promotion-de-la-sante/repertoire-des-interventions-efficaces-ou-prometteuses-en-prevention-et-promotion-de-la-sante	France
Buenas Prácticas (BBPP) en el Sistema Nacional de Salud (Collection of good practices in the National Health System in Spain)	https://www.mscbs.gob.es/organizacion/sns/planCalidadSNS/BBPP.htm	Spain

Another EU-level initiative is the Healthy Workplaces Campaigns of good practice exchange that includes an event and ceremony organised bi-annually by the European Agency for Safety and Health at Work (EU-OSHA). While demonstrating the benefits of good safety and health in the workplace, the awards also serve as a platform for sharing and promoting good practices across Europe.

There are also several national best practice portals in European countries, including database of health promotion projects in Germany, portal of health promotion interventions in the Netherlands, a database of projects and interventions in disease prevention and health promotion in Italy, portal for the exchange of examples of good practice in the field of public health in Slovenia, a database of health interventions in Poland, a portal of effective and promising interventions in health promotion and disease prevention France and collection of good practices in the National Health System in Spain. Some other countries, including Finland, are also planning to start collection of national best practices. To improve synergies, the European Commission is exploring ways of linking national and the European best practice portals, also in regards to the criteria used for their assessment, via for example, the EuroHealthNet Thematic Working Group on Health Promotion and Disease Prevention Programme Registers [7]. However, given the specific (and in some cases unique) rigorous criteria and the assessment process of best practices with the EU Public Health portal, direct linking and inclusion of practices from other portals may not be feasible at the moment.

At a global level, in 2017 WHO also published a list of “best buys” for the prevention and control of NCDs [8]. The document presents 88 interventions for the key risk factors for NCDs, namely tobacco, harmful use of alcohol, unhealthy diet and physical inactivity. The interventions, or policy options, presented in the document were identified based on demonstrated quantifiable effect size from at least one published study in a peer reviewed journal and a clear link to one of global NCDs targets, and assessed for cost-effectiveness, feasibility, as well as non-financial considerations and with special consideration for low- and middle-income countries [9]. Linked to this work, WHO Europe Compendium of good practices reports on 22 good practices in the European Region [10].

The Organisation for Economic Co-operation and Development (OECD) Health Policy Studies also include evaluation of policies on health determinants (such as obesity, harmful alcohol consumption) [11], as well policy-relevant overview of health and health systems in the EU through their Country Health Profiles [12]. It has recently published a Guidebook on Best Practices in Public Health [13].

### Establishment of criteria for selection of best practices in the EU Public Health Best Practice portal

In the context of the EU portal, a best practice is defined as “a relevant policy or intervention implemented in a real life setting which has been favourably assessed in terms of adequacy (ethics and evidence) and equity as well as effectiveness and efficiency related to process and outcomes. Other criteria are important for a successful transferability of the practice such as a clear definition of the context, sustainability, intersectorality and participation of stakeholders” [14].

The criteria for best practice selection were developed and approved in 2017 [15], in collaboration with experts from several European projects that collected good/best practices in similar fields and relevant international organisations including the WHO and the OECD. These essential criteria closely correspond to the criteria used by WHO for their best practices selection [16], as well as those discussed in the systematic review that attempted to establish a framework for selecting best practices in public health area based on 48 sources (one book, 8 peer-reviewed articles and 39 organisational sources) [4].

Experts in best practice collection and selection in the area of health promotion and chronic disease prevention and management worked on this collection of criteria and its adaptation to the purpose of pan-EU best practice collection and transfer. Before their application for the practice assessment, the draft criteria were also presented to countries’ representatives for their feedback and comments via a consultation with the Steering Group on Health Promotion, Disease Prevention and the Management of Non-Communicable Diseases (SGPP) [17]. The group consists of representatives of health ministries from the European and European Economic Area countries (EU27/EEA). As the SGPP priorities may change depending on public health situation in Europe, the assessment process is not fully fixed and may be subject to modifications. With this in mind, the criteria were updated in 2020 to be more inclusive. The first set of criteria came from the angle of NCDs and was tailored to practices in that field. The update aimed to ensure that the criteria would also fit practices in the field of infectious disease prevention and control.

### The criteria for best practice assessment

The established criteria are grouped into exclusion, core and qualifier criteria groups. Table 2 presents the groupings and individual criteria for the assessment of practices. Within each of the criteria groups, relevant sub-criteria are further considered.

**Table 2:** Criteria groupings and criteria for best practice evaluation

**• Exclusion criteria**:	
o Relevance	
o Intervention characteristics	
o Evidence and theory based	
o Ethical aspects	
**• Core criteria**:	
o Effectiveness and Efficiency of the intervention	
o Equity	
**• Qualifier criteria:**	
o Transferability	
o Sustainability	
o Participation	
o Intersectoral collaboration	

The criteria and sub-criteria are explained in detail in the document available online https://ec.europa.eu/health/sites/default/files/major_chronic_diseases/docs/sgpp_bestpracticescriteria_en.pdf

The exclusion criteria assess adequacy and completeness of the information provided. They consider the political and strategic relevance of the practice and check if such a practice is needed and addresses a valid concern. The evaluation also scores the description of the intervention, such as identification of the target population, SMART (specific, measurable, achievable, realistic and timely) objectives and methodology, as well as its theoretical basis to ascertain evidence- and theory-based approach. Ethical aspects of the practice, in terms of ensuring that the benefits outweigh harms and are equally distributed among individuals and communities, the protection of individual rights and principles and respect of autonomy, and a description of how the practice is implemented are also scored and should be thoroughly explained in the submission. Conflict of interest and clear benefits for the needs of the target group also need to be addressed. In simpler terms, these criteria assess if the current public health needs are supported by the practice, produce beneficial results for the population in need in a scientifically sound manner and are free from any commercial benefits of any individual entity.

The core criteria assess the effectiveness and efficiency of the practice, as well as how the practice has addressed equity issues. With regards to effectiveness (i.e. were the objectives achieved and measurable in a real world situation?) and efficiency (i.e. ability of achieving optimal results with the lowest possible cost), both process and outcome evaluation is essential. The process evaluation assesses the implementation of the practice that should consider social and economic aspects from both the target population and the stakeholders involved, and the type of evaluation (i.e. conducted by internal or external stakeholders). It also assesses if the outcome indicators were linked to the stated goals of the programme or intervention and if the pre- and post-intervention situation improved adequately, effectively and efficiently. The outcome evaluation assesses the expected outcome, i.e. to what extent the problem has improved. Specifically, it estimates the cost of the intervention in relation to the beneficial impact it had (cost-effectiveness) and states all obstacles that were faced during its implementation. It also validates if the evaluation outcomes were relevant to the theoretical basis and the target population. This criterion also considers equity, if the practice took into account different population groups during the implementation and adapted its scope to reduce existing health inequalities among, for example, different sexes, age, socio-economic, deprived or vulnerable groups.

The qualifier criteria evaluate transferability of the practice to other settings and contexts, its sustainability, ability of the practice to foster collaboration among different sectors and the inclusion of stakeholders through the whole cycle of the practice. The practice is scored on the extent to which the implementation results are systematized and documented, making it possible to transfer it to other contexts/settings/countries or to scale it up to a broader target population/geographic context. The documentation should include details on potential organizational and contextual elements, such as barriers (e.g. legal, managerial, financial, personal or environmental) and facilitators, as well as a communication strategy to disseminate the outcomes of the intervention. The practice should have enough institutional support and stable financial and institutional/human resources to be maintained in the long-term, taking into account socioeconomic trends. The sustainability strategy and funding should be identified. Relevant stakeholders from different sectors should be represented, fostering multidisciplinary approach and intersectoral collaboration. The practice should use means to empower and engage the community, for example capacity building or strengthening health literacy of the target population through mentoring or training.

### Best practice: from submission to the assessment process

Stakeholders, EU Member State and EEA country representatives and even citizens can submit practice proposals on the best practice portal at any time. However, there are also targeted calls published on the portal based on the priority setting of the SGPP. For the 2019 targeted call on NCDs prevention, the European Commission’s Joint Research Centre supported the SGPP by conducting a priority topic ranking exercise [18]. The process for submitting the application is as follows:

First, the submitter is required to fill in an online submission questionnaire consisting of 16 questions. Mandatory attachments are requested for some questions, notably for the detailed practice description, where submitters should explain why the practice is relevant to address the health topic it refers to and how it builds on the underlying evidence, and practice process/output evaluation details.

Second, the submitters are asked to provide all materials and responses needed in the submission for the successful evaluation of the practice. A submitters’ guide with a checklist are available to ensure that the questionnaire is filled appropriately and sufficient information for the evaluation is presented [14].

Third, the submitted practices are then assessed by a group of experts. The EC Joint Research Centre has facilitated the assessment of best practices. The practices are assessed in lots, either after a specific call or on a regular basis (most often annually) for practices submitted throughout the year. They are pre-screened and additional information may be requested from the submitters if documentation is missing or incomplete. This is done to strengthen the submission and facilitate its assessment. In general, failure to provide all required documentation or information necessary to assess all of the sub-criteria for evaluation resulted in “0” score of the criterion, considerably reducing the overall score. The examples of most commonly observed weaknesses during the assessment process are presented in Table 3. The documents can be submitted in any official EU language and in this case be the subject for translations to English using ‘EC machine translation tool’ [19].

**Table 3:** The most commonly observed weaknesses during the assessment process (in order of frequency)

1	The lack of full evaluation report (including description of process and outcome, as well as economic evaluations and/or indicators to measure the effectiveness of the practice)
2	The lack of estimation of human resources, budget and material requirements
3	No description of communication strategy
4	The lack of details on how equity and bioethical principals have been respected, ethical training of the experts and explanation how individual's rights being protected
5	No description of stakeholders involvement
6	The lack of detailed information on methodology and explanation how the practice was influenced by existing scientific evidence, conceptual frameworks, and/or approaches

For each practice, a trio of experts composed of two external evaluators and one evaluator internal to the Commission are selected based on their expertise. Gender, geographical balance, type of professional organisation and languages spoken are also considered in the selection of the evaluators to avoid conflict of interest, foster diversity and facilitate assessment of documents submitted in the original language. The internal evaluators also ensure consistency among the different evaluation trios.

The evaluators are given approximately a month to evaluate the practices remotely using the dedicated portal’s online tool, accessible only to evaluators. Once the practices are individually assessed, the trio meets online for a consensus meeting. During the consensus meeting each practice is discussed and a common score agreed for each sub criterion is agreed. One of the external evaluators acts as a rapporteur. The rapporteur moderates the discussion and prepares a qualitative consensus report agreed by the trio. The consensus report is based on comments provided for each of the criteria, as well as overall comments for the practice. Evaluators can give 0 to 10 points for each sub-criterion (Table 4).

**Table 4:** The points, rating and the description of the scoring of practices submitted to the EU best Practice portal

Points	Rating	Description
0-1	Very poor	The practice fails to address the criterion or cannot be judged due to missing or incomplete information.
2-3	Poor	The criterion is inadequately addressed, or there are serious inherent weaknesses.
4-5	Fair	The practice broadly addresses the criterion, but there are significant weaknesses.
6-7	Good	The practice addresses the criterion well, but has a few shortcomings.
8-9	Very good	The practice addresses the criterion very well, but has a few shortcomings.
10	Excellent	The practice successfully addresses all relevant aspects of the criterion. Any shortcomings are minor.

Each criteria grouping has a threshold. The evaluation is sequential, starting with the exclusion criteria. The threshold for the exclusion criteria is 128 out of 190 points. The threshold for all core criteria is 80 out of 110 points. There is also a threshold of 120 out of 180 points for all qualifier criteria together. Altogether, a practice can reach a maximum of 480 points. Only practices that pass all individual criteria thresholds summing up to 328 points (i.e. 68%) as a minimum total score are labelled as "best".

The assessment is a multilevel process. Once the best practices are identified, they are discussed among the internal Joint Research Centre evaluators and any potential conflicts of interests highlighted during the assessment are carefully checked.

Finally, the practices of which the final score oscillates around the threshold score are carefully re-visited during the internal evaluators meeting. Other Directorates General of the European Commission, responsible for relevant policy files, are informed and consulted on the final decision.

Once the assessment cycle is finalised, the submitters of the practice receive a decision letter that reflects the evaluators’ qualitative report and the intra-European Commission consultations. Submitted practices that are considered as best practices are added to the portal for potential transfer and scale up.

The whole process from the publication of a targeted call until the decision is communicated to the submitters takes on average 6 months. Since 2018 this process was applied to evaluate some hundred practices submitted to the open or targeted call on the website. Practices may be kept up to 10 years on the portal. By submitting a practice for evaluation, the submitter accepts that a summary description of the practice and their personal contact details are published on the best practice portal.

### Best practices in the portal

**Fig. 1:**
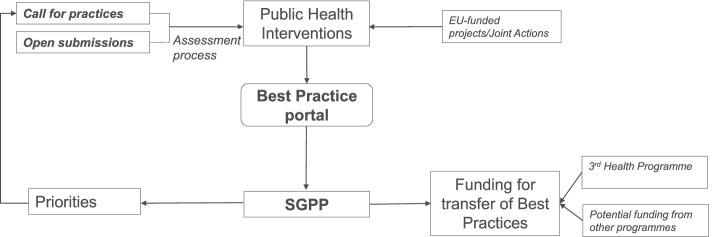
The process from selecting a best practice to funding its transfer to implement it in a different EU country

Other than the practices selected as best through the described assessment process, the portal also hosts practices selected through other processes; for example European Commission Health Programme Joint Actions, such CHRODIS - Joint Action on Chronic Diseases and Promoting Healthy Ageing across the Life Cycle (http://chrodis.eu/), JANPA - Joint Action on Nutrition and Physical Activity (http://janpa-toolbox.eu), MHCompass - EU-Compass for Action on Mental Health and Well-being (https://ec.europa.eu/health/non_communicable_diseases/mental_health/eu_compass_en), RARHA - Joint Action on Reducing Alcohol Harm (http://www.rarha.eu), SCIROCCO - Scaling Integrated Care in Context project (https://www.scirocco-project.eu/), Health Awards (https://ec.europa.eu/health/ngo_award/previous_editions_en), all ran independent evaluations and identified best or good practices that are of interest to the users of the portal and can be considered for potential transfer (Fig. 1).

The best practice portal can be fed in two ways: i) through the assessment of portal submitted practices either responding to a targeted call based on the priorities set by the European Commission and SGPP or open submission at any time; ii) fed with practices inherited from EU-funded projects or Joint Actions. The SGPP sets the priorities and facilitates implementation of practices in the EU Member States, using financial instruments available at European Commission.

SGPP- Steering Group on Health Promotion, Disease Prevention and the Management of Non-Communicable Diseases

### Transfer of best practices across the EU

Naturally, every user of the portal is free to consult the summary description of the practices and contact the submitters for further information and potential collaboration. In addition to this, the European Commission has put in place a structured process to fund the transfer of selected practices. The SGPP plays a crucial role in this regard. Not only it prioritises the topics for targeted calls for best practices [18], but also selects those for implementation in their countries. During dedicated marketplace workshops, selected practices are presented by the submitter, or the practice responsible person, to interested countries’ representatives. Until now five marketplaces took place, of which the last one was held online. The details for the marketplaces can be found online [20]. The representatives are then invited to choose those practices that they wish to transfer and implement in their own countries.

Since 2017, fourteen best practices have been implemented in several interested countries with the total European Commission investment of 30 million Euros. Other 17 million Euros are foreseen to transfer the nine best practices selected in 2021. More details can be found on the website: https://webgate.ec.europa.eu/dyna/bp-portal/transferred.cfm. Figure 1 illustrates the process from best practice identification to implementation. The ultimate goal of the portal is not only to identify and disseminate best practices in public health, but to assure the implementation and transfer of practices to improve the health of people. The Commission is also exploring how to promote promising interventions that have not been implemented yet in real life settings. This will allow their parallel evaluation and implementation.

The European Commission is also setting up a new EU NCD Initiative. One of its aims is to facilitate transfer of evidence-based good and promising practices on health promotion and disease prevention between the EU Member States.

## Conclusion

The exchange of best practices in the area of health is a promising strategy for tackling NCDs and is more and more recognised by national and international public health bodies, given the existence of several best practice portals both in Europe and worldwide. Successful transfer of best practices between European countries can be an important contribution to reaching the relevant Sustainable Development Goals and may also trigger countries to increase their spending on disease prevention, which currently oscillates at a level below 5% in Europe [21].

The European Commission is taking this concept much further – moving from mere information on “what works” to financially supporting the actual transfer of a best practice to other EU countries. Through this, it can support national disease prevention and health promotion policies. The EU Public Health Best Practice portal is a key tool to support this process. Policy makers can find best evidence on the portal and through the whole process shall obtain support for implementation of effective interventions within the EU to protect their population.

In conclusion, moving from a mere sharing and exchange of best practices to actual transfer to other countries is an important tool that helps to bridge the gap between public health challenges and practical solutions.

## Data Availability

All material is available on the Best Practice portal: https://webgate.ec.europa.eu/dyna/bp-portal/.

## Abbreviations

EU: European Union
NCDs: Non-communicable diseases
EMCDDA: European Monitoring Centre for Drugs and Drug Addiction
EU-OSHA: European Agency for Safety and Health at Work
WHO: World Health Organisation
OECD: Organisation for Economic Co-operation and Development
EEA: European Economic Area European Economic Area
SGPP: Steering Group on Health Promotion, Disease Prevention and the Management of Non-Communicable Diseases
